# Methylation of *WT1*, *CA10* in peripheral blood leukocyte is associated with breast cancer risk: a case-control study

**DOI:** 10.1186/s12885-020-07183-8

**Published:** 2020-07-31

**Authors:** Anqi Ge, Song Gao, Yupeng Liu, Hui Zhang, Xuan Wang, Lei Zhang, Da Pang, Yashuang Zhao

**Affiliations:** 1 Department of Epidemiology, School of Public Health, Harbin Medical University, 57 Baojian Street, Nangang District, Harbin, 150081 Heilongjiang Province People’s Republic of China; 2 Department of Breast Surgery, the Tumor Hospital of Harbin Medical University, 150 Haping Street, Nangang District, Harbin, 150081 Heilongjiang Province People’s Republic of China

**Keywords:** Breast cancer, *CA10*, *WT1*, DNA methylation, Leukocytes

## Abstract

**Background:**

Studies have shown that abnormal changes of specific-gene DNA methylation in leukocytes may be associated with an elevated risk of cancer. However, associations between the methylation of the zinc-related genes, *WT1* and *CA10*, and breast cancer risk remain unknown.

**Methods:**

The methylation of *WT1* and *CA10* was analyzed by methylation-sensitive high-resolution-melting (MS-HRM) in a case-control study with female subjects (*N* = 959). Logistic regression was used to analyze the associations, and propensity score (PS) method was used to adjust confounders.

**Results:**

The results showed that *WT1* hypermethylation was associated with an increased risk of breast cancer, with an odds ratio (OR) of 3.07 [95% confidence interval (CI): 1.67–5.64, *P* < 0.01]. Subgroup analyses showed that *WT1* hypermethylation was specifically associated with an elevated risk of luminal A subtype (OR = 2.62, 95% CI: 1.11–6.20, *P* = 0.03) and luminal B subtype (OR = 3.23, 95% CI: 1.34–7.80, *P* = 0.01). *CA10* hypermethylation was associated with an increased risk of luminal B subtype (OR = 1.80, 95% CI: 1.09–2.98, *P* = 0.02).

**Conclusion:**

The results of the present study suggest that the hypermethylation of *WT1* methylation in leukocytes is significantly associated with an increased risk of breast cancer. The hypermethylation of *WT1* is associated with an increased risk of luminal subtypes of breast cancer, and the hypermethylation of *CA10* is associated with an increased risk of luminal B subtype of breast cancer.

## Background

Breast cancer is one of the most common malignancies in women worldwide [1] and presents with different molecular subtypes, including luminal A, luminal B, HER2-enriched, and basal-like that also called triple negative [2]. As a major type of epigenetic modification, DNA methylation is involved in regulating cellular processes, including chromosomal instability [3] and gene expression. The hypermethylation of CpG regions in specific genes contribute to neoplastic formation through the transcriptional silencing of tumor suppressor genes. Aberrant patterns of specific gene methylation can help identifying differences in breast cancer subtypes [2], and showing promise for utilizing in large-scale epidemiological studies. It has been suggested that leukocyte DNA methylation, as a simple non-invasive blood marker [4, 5], could serve as a surrogate for systematic methylation activity and offers great potential for predicting the increased risk of breast cancer [6].

Wilm’s Tumor gene (*WT1*) is a tumor suppressor gene which involved in human cell growth and differentiation. *WT1* has been reported to be significantly different methylated in the tissues of hepatocellular carcinoma [7], lung cancer [8] and breast cancer [9]. *WT1* aberrant methylation may lead to a reduction or absence of *WT1* expression, which results in the overexpression of the insulin-like growth factor I receptor (IGF 1R) and insulin-like growth factor II (IGF II), thereby promoting breast cancer process [10–12]. CA10 is a member of the carbonic anhydrase family, which is a large family of zinc-containing metalloenzymes that catalyze the reversible hydration of carbon dioxide and the dehydration of carbonic acid [13]. Ivanov et al. suggested that the induction or enhancement of carbonic anhydrase expression may contribute to the tumor microenvironment by maintaining an extracellular acidic pH and helping the growth and metastasis of cancer cells [14]. Studies have demonstrated that the abnormal expression of carbonic anhydrase family by aberrant methylation is related with gastric cancer and the metastasis of ovary tumors [13, 15]. Furthermore, Wojdacz et al. reported that both *WT1* and *CA10* hypermethylation were significantly different between breast cancer tumor tissues and non-malignant tissues [16]. However, how the methylation of these two genes in leukocyte DNA affects breast cancer susceptibility remains unclear.

In this study, we investigated the associations between the methylation of *WT1*, *CA10* in peripheral blood leukocyte DNA and breast cancer risk. We subsequently used an external dataset of a nested case-control cohort within the EPIC-Italy cohort study as external data to validate the association between gene methylation and breast cancer risk. We also investigated the associations between the methylation of these two genes and the risk of different molecular types of breast cancer.

## Methods

### Study subjects

We investigated the relationship between *WT1* and *CA10* methylation and breast cancer risk using a case-control study. All the included breast cancer patients were newly diagnosed females and were recruited from the Tumor Hospital of Harbin Medical University from 2010 to 2014. Female breast cancer subjects were included if they diagnosed with invasive ductal carcinoma (IDC) or ductal carcinoma in situ (DCIS), other types of breast cancer (such as lipoma of the breast, metastatic breast cancer, etc.) were excluded from our study. Controls were recruited from patients admitted to the Orthopedic and Ophthalmology Department of the Second Affiliated Hospital of Harbin Medical University and volunteers from the Xiangfang community of Harbin within the same period. All controls were also female. In addition, all control participants were asked about their disease history in a questionnaire, and individuals who reported a history of any cancer were excluded from our final subjects. Finally, 402 female breast cancer cases and 557 female controls were included in our study. Blood sample (5 mL) was collected from each participant and then stored at − 80 °C.

### Data collection

All subjects were interviewed face-to-face by trained investigators with normalized questioning methods. The questionnaire was adopted from the study by Shu et al. [17], and included information on demographic information (age, ethnicity, and others); daily dietary intake (vegetables, fruits, beverages, and snacks); behaviors (smoking, drinking, physical activity and work activity); female-specific questions involving menstruation status, breast disease history (lobular hyperplasia, cyst, and others); gynecologic surgery history (hysterectomy, ovariotomy) and family history of cancer and breast cancer. The questions involved in dietary and behavioral were about the participants’ daily routine of 1 year prior to the interview. The basic demographic characteristics and environmental factors of the study subjects are presented in Table S1.

**Fig. 1:**
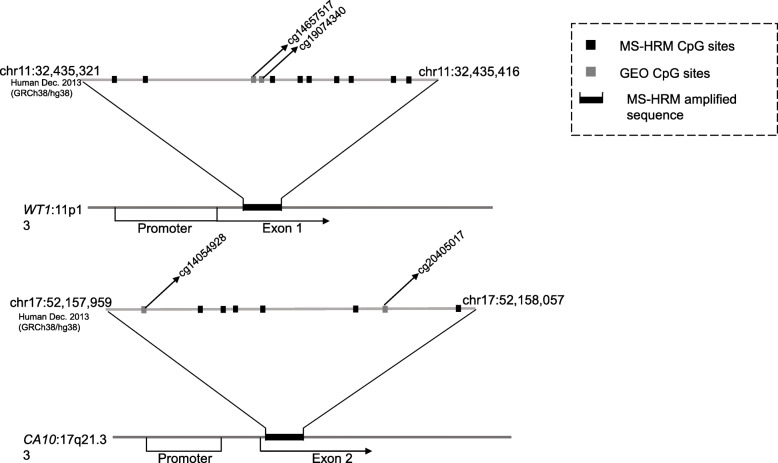
MS-HRM amplified sequence of *WT1* and *CA10* and the validated Cg sites in GSE51032

The study was validated with the GEO-GSE51032 (IPEC-Italy cohort) dataset with a nested case control study design to analyze the association between the methylation of *CA10* and *WT1* and breast cancer risk. The blood samples were also collected and other anthropometric measurements were taken. The sample selection criteria and the methods were reported by Riboli et al. [18]. We extracted all 232 female breast cancer cases and all 340 female controls from this nested case-control study and located the methylation probes from the Illumina 450 K array. The annotated CG sites covered by our MS-HRM sequence are illustrated in Fig. 1.

### DNA extraction and bisulfite conversion

DNA was extracted from peripheral blood samples using a commercial DNA extraction kit (QIAamp DNA Blood Mini Kit, Hilden, Germany). The concentration and the purity of DNA were assessed using a Nanodrop 2000 Spectrophotometer (Thermo Scientific, USA). Genomic DNA was bisulfite-modified with an EpiTect Bisulfite kit (Qiagen, Hilden, Germany). Bisulfite DNA was normalized to a concentration of 20 ng/mL and was stored at − 20 °C for the following experiment. DNA extraction and DNA sodium bisulfite modification were performed according to the manufacturers’ instructions.

### Gene methylation status analysis

**Fig. 2:**
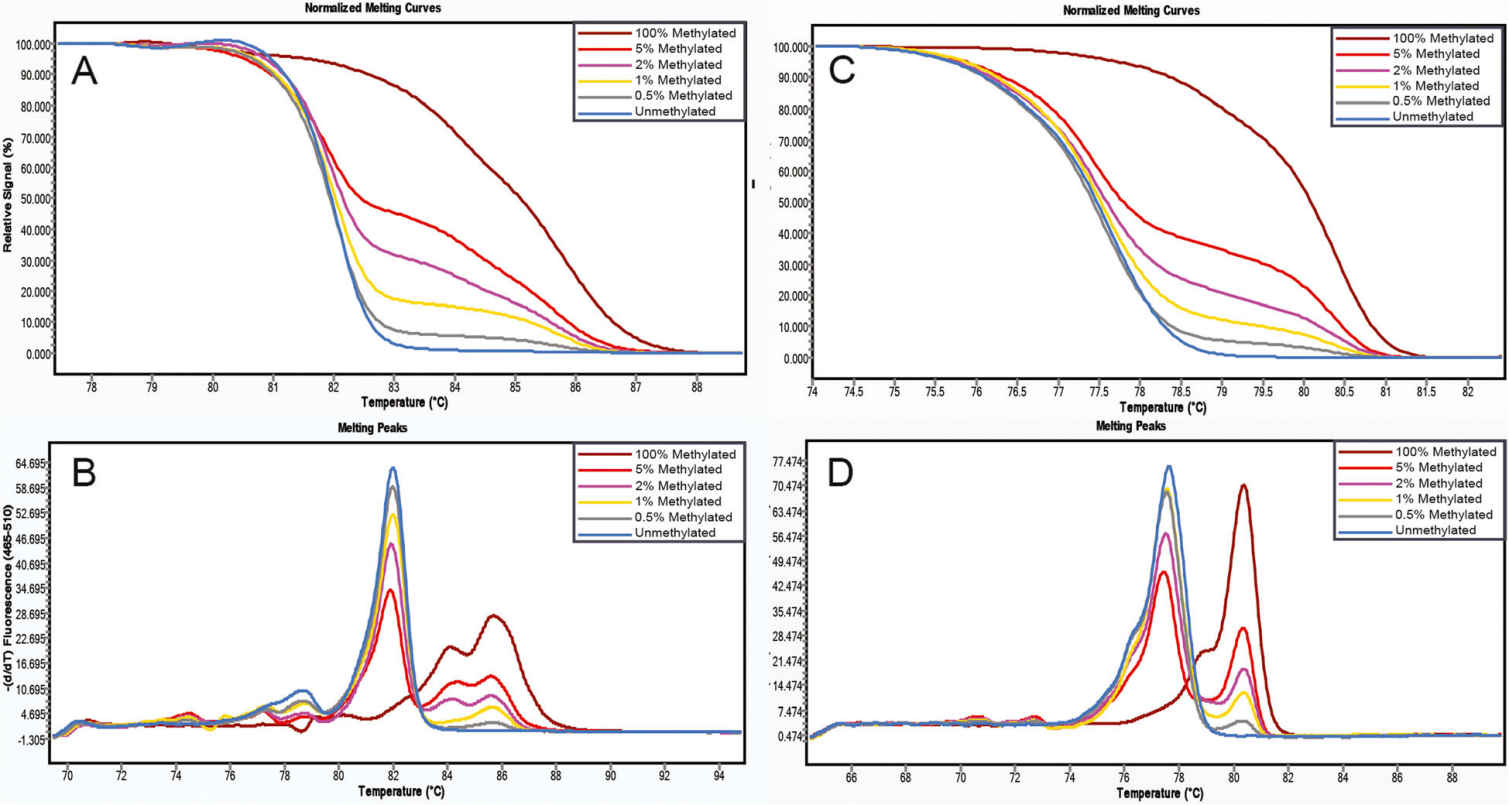
The MS-HRM based method for *WT1* and *CA10* methylation detection. The figures showed normalized melting curves and melting peaks for standards methylation level and of *WT1*(A)(B) and *CA10*(C)(D).The methylation status of the standards were 0, 0.5, 1, 2, 5, 100%, respectively

We performed methylation-sensitive high-resolution melting analysis (MS-HRM) to evaluate the methylation of *WT1* and *CA10* with the LightCycler 480 system (Roche Applied Science, Mannheim, Germany) equipped with Gene Scanning software (version 2.0). The primers were adopted from a published study [16]. We used universal methylated and unmethylated DNA standards (ZYMO, USA) and mixed them at different ratios to create standards with a 0.5, 1, 2, and 5% methylation levels of *WT1* and *CA10* (Fig. 2). PCR amplification and MS-HRM were optimized and performed. The conditions, reaction mixture and primer sequences used in the MS-HRM experiments are listed in Table S2–3. Each standard reaction was performed in duplicate in each run. Each plate included duplicate water blanks as negative controls. We also repeated some samples in different runs to assess the consistency of the experiment. There was a significant agreement of these samples in different runs with respect to the observed methylation status of *WT1* and *CA10,* with kappa value of 1.00 (*P* < 0.01) and 0.94 (*P* < 0.01), respectively (Table S4).

### Definitions of different molecular subtypes of breast cancer

Four subtypes of breast cancer were defined as luminal A, luminal B, HER-2 enriched and triple negative breast cancer (TNBC) by immunohistochemical analysis based on previously validated clinicopathological criteria [19].

### Statistical analysis

For the distribution of basic demographic characteristics, continuous variables such as age were analyzed by two-sample t-tests, and categorical variables were analyzed by chi-square (χ^2^) tests. For missing values in the environmental factors, we applied multiple imputation to generate possible values. To measure the association between methylation of *WT1*, *CA10* and breast cancer risk and different molecular types breast cancer, we used univariate and multivariate unconditional logistic regression analyses to estimate the crude and adjusted odds ratios (ORs) and 95% confidence intervals (95% CIs). For our case-control study, we used 0% methylation as a cutoff for both *WT1* and *CA10.* We used receiver operating characteristic curve (ROC) to calculate the cut-off value of β for the validation dataset. We also applied the propensity score (PS) method to adjust covariates (involving all environmental factors in the questionnaire), in which the study outcome served as the dependent variable and PS served as the confounding variable. Kappa values were calculated to analyze the consistency between same samples in different runs. All two-sided *P* values < 0.05 were considered statistically significant. Data were analyzed by using SPSS v.24.0 (SPSS Inc., Chicago, IL, USA).

## Results

### Characteristics of the cases and controls

This study included 402 female cases with a mean age of 51.75 ± 9.39 and 557 female controls with a mean age of 51.85 ± 10.31. Other demographic information of the cases and controls is listed in Table 1. The definition of variables for environmental factors with ≤5.8% missing data were processed by the multiple imputation method are presented in Table S1.

**Table 1:** Demographic characteristics of breast cancer patients and controls

Characteristics	No. of Controls(%)	No. of Cases (%)	*P* Value
Age			
Mean ± SD	51.85 ± 10.31	51.75 ± 9.39	
< 40	82(14.7)	41(10.2)	0.02
40-	333(59.8)	274(68.2)	
≥ 60	142(25.5)	87(21.6)	
BMI			
≤ 18.5	35(6.3)	14(3.5)	0.12
18.5-	274(49.2)	211(52.5)	
≥ 24.0	248(44.5)	177(44.0)	
Urban and Rural Status			
Rural	236(42.4)	232(57.7)	< 0.01
Urban	321(57.6)	170(42.3)	
Education Level			
Primary School or Below	162(29.1)	98(24.4)	0.27
Middle School	175(31.4)	135(33.6)	
Senior School and Higher	220(39.5)	169(42.0)	
Occupation Type ^a^			
White Collar	273(49.0)	233(58.2)	0.01
Blue Collar	284(51.0)	169(41.8)	
Ethnicity			
Han	529(95.0)	386(96.0)	0.27
Other	28(5.0)	16(4.0)	

^a^ The white collar occupation referred to people work that need mental rather than physical effort, such as office, doctor, accountant, business, teacher, etc.; the blue collar occupation referred to people work as manual labor, such as worker, farmer, cleaner, etc.

### Associations between *WT1*, *CA10* methylation and breast cancer risk

*WT1* methylation was associated with breast cancer risk both in multivariable and PS adjusted methods with ORs of 2.42 (95% CI: 1.45–4.04, *P* < 0.01) and 3.07 (95% CI: 1.67–5.64, *P* < 0.01), respectively. *CA10* methylation was statistically significant associated with breast cancer in the multivariable adjustment with an OR of 1.53 (95% CI: 1.14–2.05, *P* < 0.01), but was only marginally associated with breast cancer after PS adjustment, with an OR of 1.35 (95% CI: 0.97–1.90, *P* = 0.08) (Table 2).

**Table 2:** The associations between gene methylation and risk of breast cancer and different molecular types of breast cancer

		Molecular types ^a^	No. of Unmethylation(%)	No. of Methylation(%)	Crude OR (95% CI)	*P* Value	OR_adjusted_^b^ (95% CI)	*P* Value	OR_adjusted_^c^ (95% CI)	*P* Value
*WT1*	Control		65(11.7)	492(88.3)	1		1		1	
		Luminal A	9(6.4)	132(93.6)	1.99(0.94-4.23)	0.07	2.61(1.18-5.74)	0.02	2.62(1.11-6.20)	0.03
		Luminal B	8(6.0)	125 (94.0)	2.12(1.50-2.99)	0.07	2.49(1.13-5.51)	0.02	3.23(1.34-7.80)	0.01
		HER-2 Enriched	5(8.9)	51(91.1)	1.34(0.51-3.50)	0.55	1.91(0.69-5.30)	0.21	1.91(0.66-5.51)	0.23
		TNBC	1(2.9)	33(27.1)	4.34(0.58-32.33)	0.15	5.63(0.73-43.63)	0.10	6.04(0.76-47.90)	0.09
	All cases		26(6.5)	376(93.5)	1.92(1.18-3.13)	0.01	2.42(1.45-4.04)	0.01	3.07(1.67-5.64)	<0.01
*CA10*	Control		209(37.5)	348(62.5)	1		1		1	
		Luminal A	40(28.4)	101(71.6)	1.52(1.00-2.26)	0.05	1.60(1.04-2.45)	0.03	1.51(0.94-2.41)	0.09
		Luminal B	34(25.6)	99(74.4)	1.79(1.17-2.74)	0.01	2.04(1.30-3.21)	<0.01	1.80(1.09-2.98)	0.02
		HER-2 Enriched	18(32.1)	38(67.9)	1.27(0.71-2.29)	0.43	1.42(0.76-2.66)	0.27	1.37(0.71-2.63)	0.35
		TNBC	14(41.1)	20(58.8)	0.86(0.43-1.74)	0.67	0.94(0.45-1.96)	0.87	1.01(0.46-2.20)	0.99
	All cases		119(29.6)	283(70.4)	1.43(1.08-1.88)	0.01	1.53(1.14-2.05)	<0.01	1.35(0.97-1.90)	0.08

^a^The result excluded 38 breast cancer patients with incomplete immunohistochemical records

^b^Adjusted for age, BMI, ethnicity, urban and rural status and family history of breast cancer and cancer

^c^Adjusted by propensity score(potential confounder included age, BMI, urban and rural status, ethnicity, education level, mammography, gynecologic surgery, breast disease history, menstrual cycle, menopause, reproduction, abortion, breast feeding, oral contraceptive, female hormone intake, fruit intake, vegetable intake, tomato intake, broccoli intake, bean products, pungent food, pork, beef and lamb consumption, chicken consumption, sea-fish, egg, diary, fungus, pickles, alcohol consumption, tea consumption, cigarette, physical activity, occupation type, family history of breast cancer and cancer)

In the subgroup analyses, after PS adjustment, *WT1* methylation was associated with breast cancer risk in both the younger (< 60-years-old) and older (≥60-years-old) groups, with ORs of 2.64 (95% CI: 1.31–5.32, *P* = 0.01) and 4.72 (95% CI: 1.31–16.97, *P* = 0.01), respectively. *CA10* methylation was associated with breast cancer risk in younger age group (< 60-years-old) before PS adjustment, with OR of 1.56 (95% CI: 1.15–2.11, *P* = 0.01); However, the association was not statistically significant after PS adjustment (Table 3). We also analyzed the combination and interaction of age and the methylation of *WT1*, *CA10* on the risk of breast cancer. The *P* values for the interactions between age and the methylation of *WT1* and *CA10* on the risk of breast cancer were 0.40 and 0.73, respectively. The results are presented in Table 4.

**Table 3:** The subgroup analysis of the associations between methylation of genes and the risk of breast cancer based on different age

	Crude OR (95% CI)	*P* Value	OR _adjusted_^a^ (95% CI)	*P* Value
*WT1*				
<60				
Unmethylation	1		1	
Methylation	1.64(0.95–2.84)	0.08	2.64(1.31–5.32)	0.01
≥ 60				
Unmethylation	1		1	
Methylation	3.16(1.05–9.50)	0.04	4.72(1.31–16.97)	0.01
*CA10*				
<60				
Unmethylation	1		1	
Methylation	1.56(1.15–2.11)	0.05	1.32(0.90–1.96)	0.15
≥ 60				
Unmethylation	1		1	
Methylation	1.20(0.61–2.37)	0.60	1.52(0.69–3.37)	0.30

^a^ Adjusted by propensity score

**Table 4:** The interaction between age and gene methylations on the risk of breast cancer

	Age			
	≥60	< 60	Interaction	*P*
	OReg_adjusted_^a^ (95% CI)		ORi_adjusted_^a^ (95% CI)	
*WT1*				
Unmethylation	1	1.70(0.40–6.84)	1	
Methylation	4.90(1.36–17.67)	4.44(1.29–15.34)	0.53(0.13–2.28)	0.40
*CA10*				
Unmethylation	1	1.17(0.54–2.54)	1	
Methylation	1.55(0.70–3.45)	1.55(0.74–3.27)	0.86(0.35–2.09)	0.73

^a^ Adjusted for propensity score

### Associations between methylation of *WT1*, *CA10* and risk of different molecular types of breast cancer

*WT1* methylation was significantly associated with the risk of luminal A subtype of breast cancer with multivariable adjusted OR of 2.61 (95% CI: 1.18–5.74, *P* = 0.02), and PS adjusted OR of 2.62 (95% CI: 1.11–6.20, *P* = 0.03). *WT1* methylation was also significantly associated with the risk of luminal B subtype breast cancer with ORs of 2.49 (95% CI: 1.13–5.51, *P* = 0.02) and 3.23 (95% CI: 1.34–7.80, *P* = 0.01) after multivariable and PS adjustment. However, *WT1* methylation was not significantly associated with the risk of HER-2 enriched and TNBC subtypes (Table 2).

The associations between *CA10* methylation and the risk of luminal B subtype breast cancer with multivariable adjusted and PS adjusted ORs were 2.04 (95% CI: 1.30–3.21, *P P* < 0.01) and 1.80 (95% CI: 1.09–2.98, *P* = 0.02), respectively. However, *CA10* methylation had no significant associations with the risk of luminal A, HER-2 enriched and TNBC subtypes after the adjustment of PS. The association between the methylation of *WT1*, *CA10* and other clinicopathological characteristics of breast cancer patients were analyzed are showed in Table S5.

### Association between *WT1*, *CA10* methylation and breast cancer risk in GEO dataset

The GSE51032 dataset is a nested case control study that includes 233 female breast cancer cases and 340 female cancer-free controls. After the data extraction from the 450 K array, we identified two CG loci each in our targeted *WT1* and *CA10* sequences (Fig. 1). ROC curves were used to calculate the cut-off values of β, which were 0.057 and 0.226 for average β of probes in *WT1* and *CA10*. The average methylation level of Cg14657517 and Cg19074340, which are located within the *WT1* targeted sequence, was associated with breast cancer with OR of 1.88 (95% CI: 1.25–2.83, *P* = 0.03). However, the average methylation level of Cg14054928 and Cg20405017, which are located within the targeted *CA10* sequence, was not statistically significant associated breast cancer risk (OR = 0.76, 95% CI: 0.54–1.06, *P* = 0.11) (Table 5).

**Table 5:** The association between gene average CpG sites methylation and risk of female breast cancer in GEO51032

	Hypomethylation(%)	Hypermethylation (%)	Crude OR (95% CI)	*P* Value
*WT1*				
Control	285(83.8)	55(16.2)	1	
Case	171(73.4)	62(26.6)	1.88(1.25–2.83)	0.03
*CA10*				
Control	146(42.9)	194(57.1)	1	
Case	116(49.8)	117(50.2)	0.76(0.54–1.06)	0.11

## Discussion

This is the first case-control study to investigate the associations between the methylation of *WT1*, *CA10* in leukocyte DNA and breast cancer risk, and the risk of different molecular subtypes of breast cancer in a Chinese female population. After PS adjustment, we observed that methylation of *WT1* was significantly elevated breast cancer risk by 2.07-fold, *CA10* methylation was marginally associated with breast cancer risk with OR of 1.35. Women with *WT1* methylation presented a 1.62 higher risk of luminal A and 2.23 higher risk of luminal B subtype of breast cancer than those without methylation. *CA10* methylation was significantly associated with the risk of luminal B subtype with OR of 1.80. We subsequently used GEO-GSE51032 dataset, a nested case control study with clear temporal relationship between methylation changes and breast cancer, as an external dataset to validate our retrospective study. The nested case control study’s results showed a lower but still significant association between *WT1* methylation and breast cancer risk, but the association between *CA10* methylation and breast cancer risk was not statistically significant.

Breast cancer is a heterogeneous disease with different molecular subtypes, which may present different genetic and epigenetic susceptibilities. Previous studies predominantly focused on the aberrant methylation in tissue samples and its association with the risk of different molecular types of breast cancer [20, 21], with few studies having focused on the gene-specific methylation in leukocyte DNA. The methylation alternation in leukocyte DNA presented a response of the hematopoietic system [22]. Leukocyte DNA methylation can represent germline methylation, which can be used to analyze the association with cancer risk [23]. It was further reported that *BRCA1* hypermethylation in peripheral blood DNA was associated with TNBC with an OR of 5.0 [24]. The results of our study indicated that after PS adjustment, *WT1* methylation was associated with the risk of luminal A and luminal B subtypes of breast cancer with ORs of 2.62 and 3.23, and *CA10* methylation was significantly associated with luminal B subtype of breast cancer with OR of 1.80.

*WT1* is a zinc finger transcription factor located on 11p13, which was first identified as a tumor suppressor gene. *WT1* exon displayed significantly increased methylation in cancer tissue compared to nonmalignant breast tissue [16]. *WT1* methylation in the promoter and first exon region was shown to be associated with the silencing of *WT1* mRNA expression in MCF-7 and MDA-MB-231 breast cancer cells [9]. Our investigated sequence was 160 bp downstream of the Laux et al. sequence position. Here, we observed methylation of the CpG island in the first exon of *WT1* in blood leukocyte DNA, which contains 11 CpGs in the CpG island. Furthermore, we used external data from an IPEC- Italy cohort (GEO-GSE51032) with a nested case control study design and found the significant association between *WT1* methylation and breast cancer risk, with two CpG probes inside our sequence, with OR of 1.88.

A previous study showed *CA10* can undergo methylation during breast carcinogenesis in tumor tissue [16]. *CA10* was reported to be hypermethylated among a panel of genes in urine, which may contribute to the highly accurate and early detection of bladder cancer [25]. The result of our study suggested that *CA10* methylation in leukocyte DNA was marginally associated with an elevated breast cancer risk after PS adjustment. The amplified sequence contained 7 CpGs and located in the second exon of *CA10*. The external validation dataset of GEO-GSE51032 only included 2 CpG probes and did not exhibit a significant association between *CA10* hypermethylation and breast cancer risk.

To further investigate the functional relevance of the observed associations, it would be important to test whether methylation of the specific CpGs in *WT1* and *CA10* associated with the alteration of their expression. Therefore, we investigated the correlations between methylation probes and expression using TCGA (http://cancergenome.nih.gov/) and Mexpress (https://mexpress.be/) databases. The results showed that *WT1* hypermethylation was also negatively correlated with its expression (Cg14657517, *r* = − 0.204, *P* < 0.001; Cg19074340, *r* = − 0.201, *P* < 0.001), and *CA10* hypermethylation was negatively related to its mRNA expression as well (Cg14054928, *r* = − 0.182, *P* < 0.001; Cg20405017, *r* = − 0.162, *P* < 0.001). Although discounted by different sample-derived DNA, the significant negative correlations between *WT1*, *CA10* methylation and gene expression were consistent with our study and indicated promising potential in breast cancer risk assessment.

In our previous study, we tested the accuracy of MS-HRM by assessing the *WT1* methylation level with both MS-HRM and pyrosequencing, and the results were highly correlated between these two methods [26]. However, the methylation level of leukocyte DNA is relatively low and the limitation of pyrosequencing is 2%. As a reliable and highly sensitive technique, MS-HRM can be used to assess the methylation level of targeted CpGs as low as 0.1% [27]. The high consistency of our results for different runs which making the non-misclassification of methylation level between case and control and the probability of higher sensitivity of MS-HRM comparing pyrosequencing can make our study results more conserved [28].

The limitations of this study should be taken into consideration before drawing a conclusions: first, as in all retrospective analyses, our study may have some recall bias when collecting information on environmental factors. Second, the sample size of our study is not large enough for subgroup analysis, including the subgroup analyses of low frequency environmental factors, such as smoking behavior, therefore their associations with DNA methylation of *WT1, CA10* could not be analyzed. Third, selection bias may have occurred, since we recruited the breast cancer patient subjects at the Tumor Hospital of Harbin Medical University, which might not be representative of the distribution of breast cancer patients to some extent.

## Conclusion

In summary, the results of our study suggested that methylation of *WT1*, *CA10* in blood leukocytes may be associated with the risk of breast cancer. Associations between *WT1* methylation and the risk of luminal subtypes of breast cancer and between *CA10* methylation and the risk of luminal B subtype breast cancer were also observed.

## Supplementary information

**Additional file1 Table S1.** Demographic variables and questionnaire-derived variables of participants before and after multiple imputation in this study.

**Additional file2 Table S2.** Primer sequences and reaction condition for methylation-sensitive high-resolution melting analysis.

**Additional file3 Table S3.** Reaction system for methylation-sensitive high-resolution melting analysis of *WT1* and *CA10.*

**Additional file4 Table S4.** Result of methylation-sensitive high-resolution melting analysis for the same samples in different runs.

**Additional file5 Table S5.** The methylation of *WT1* and *CA10* and clinicopathological characteristics in breast cancer patients.

## Data Availability

The datasets used and/or analysed during the current study are available from the corresponding author on reasonable request.

## Abbreviations

*WT1*: Wilm’s tumor 1
*CA10*: Carbonic anhydrase 10
IDC: Invasive ductal carcinoma
DCIS: Ductal carcinoma in situ
MS-HRM: Methylation-sensitive high-resolution melting
TNBC: Triple negative breast cancer
CIs: Confidence intervals
OR: Odds ratio
ROC: Receiver operating characteristic curve
ER: Estrogen receptor
PR: Progesterone receptor
HER-2: Human epidermal growth factor receptor-2
BMI: Body mass index

## References

[1] Bray F, Ferlay J, Soerjomataram I, Siegel RL, Torre LA, Jemal A (2018). Global cancer statistics 2018: GLOBOCAN estimates of incidence and mortality worldwide for 36 cancers in 185 countries. CA Cancer J Clin.

[2] Kamalakaran S, Varadan V, Giercksky Russnes HE, Levy D, Kendall J, Janevski A, Riggs M, Banerjee N, Synnestvedt M, Schlichting E (2011). DNA methylation patterns in luminal breast cancers differ from non-luminal subtypes and can identify relapse risk independent of other clinical variables. Mol Oncol.

[3] Esteller M (2007). Cancer epigenomics: DNA methylomes and histone-modification maps. Nat Rev Genet.

[4] Kang HJ, Kim JM, Kim SY, Kim SW, Shin IS, Kim HR, Park MH, Shin MG, Yoon JH, Yoon JS (2015). A longitudinal study of BDNF promoter methylation and depression in breast Cancer. Psychiatry Investig.

[5] Woo HD, Kim J (2012). Global DNA Hypomethylation in peripheral blood leukocytes as a biomarker for Cancer risk: a meta-analysis. PLoS One.

[6] Guan Z, Yu H, Cuk K, Zhang Y, Brenner H (2019). Whole-blood DNA methylation markers in early detection of breast Cancer: a systematic literature review. Cancer Epidemiol Biomark Prev.

[7] Mžik M, Chmelařová M, John S, Laco J, Slabý O, Kiss I, Bohovicová L, Palička V, Nekvindová J (2016). Aberrant methylation of tumour suppressor genes WT1, GATA5 and PAX5 in hepatocellular carcinoma. Clin Chem Lab Med.

[8] Bruno P, Gentile G, Mancini R, Vitis CD, Esposito MC, Scozzi D, Mastrangelo M, Ricci A, Mohsen I, Ciliberto G (2012). WT1 CpG islands methylation in human lung cancer: a pilot study. Biochem Biophys Res Commun.

[9] Laux DE, Curran EM, Welshons WV, Lubahn DB, Huang THM (1999). Hypermethylation of the Wilms' tumor suppressor gene CpG island in human breast carcinomas. Breast Cancer Res Treat.

[10] Werner H, Re GG, Drummond IA, Sukhatme VP, Rd RF, Sens DA, Garvin AJ, Leroith D, Roberts CT (1993). Increased expression of the insulin-like growth factor I receptor gene, IGF1R, in Wilms tumor is correlated with modulation of IGF1R promoter activity by the WT1 Wilms tumor gene product. Proc Natl Acad Sci U S A.

[11] Paik S (1992). Expression of IGF-I and IGF-II mRNA in breast tissue. Breast Cancer Res Treat.

[12] Silberstein GB, Horn KV, Strickland P, Roberts CT, Daniel CW (1997). Altered expression of the WT1 Wilms tumor suppressor gene in human breast cancer. Proc Natl Acad Sci U S A.

[13] Nakamura J, Kitajima Y, Kai K, Hashiguchi K, Hiraki M, Noshiro H, Miyazaki K (2011). Expression of hypoxic marker CA IX is regulated by site-specific DNA methylation and is associated with the histology of gastric Cancer. Am J Pathol.

[14] Ivanov S, Liao SY, Ivanova A, Danilkovitch-Miagkova A, Tarasova N, Weirich G, Merrill MJ, Proescholdt MA, Oldfield EH, Lee J (2001). Expression of hypoxia-inducible cell-surface transmembrane carbonic anhydrases in human cancer. Am J Pathol.

[15] Sung HY, Ju W, Ahn JH (2014). DNA hypomethylation-mediated overexpression of carbonic anhydrase 9 induces an aggressive phenotype in ovarian cancer cells. Yonsei Med J.

[16] Wojdacz TK, Windeløv JA, Thestrup BB, Damsgaard TE, Overgaard J, Hansen LL (2014). Identification and characterization of locus-specific methylation patterns within novel loci undergoing hypermethylation during breast cancer pathogenesis. Breast Cancer Res.

[17] Shu XO, Yang G, Jin F, Liu D, Kushi L, Wen W, Gao YT, Zheng W (2004). Validity and reproducibility of the food frequency questionnaire used in the Shanghai Women's health study. Eur J Clin Nutr.

[18] Riboli E, Hunt KJ, Slimani N, Ferrari P, Norat T, Fahey M, Charrondiere UR, Hemon B, Casagrande C, Vignat J (2002). European prospective investigation into Cancer and nutrition (EPIC): study populations and data collection. Public Health Nutr.

[19] Coates AS, Winer EP, Goldhirsch A, Gelber RD, Gnant M, Piccart-Gebhart M, Thurlimann B, Senn HJ, Panel M (2015). Tailoring therapies--improving the management of early breast cancer: St Gallen international expert consensus on the primary therapy of early breast Cancer 2015. Ann Oncol.

[20] Conway K, Edmiston SN, May R, Pei FK, Chu H, Bryant C, Tse CK, Swift-Scanlan T, Geradts J, Troester MA (2014). DNA methylation profiling in the Carolina breast Cancer study defines cancer subclasses differing in clinicopathologic characteristics and survival. Breast Cancer Res Bcr.

[21] Holm K, Hegardt C, Staaf J, Vallonchristersson J, Jönsson G, Olsson H, Borg Å, Ringnér M (2010). Molecular subtypes of breast cancer are associated with characteristic DNA methylation patterns. Breast Cancer Res.

[22] Koestler DC, Marsit CJ, Christensen BC, Accomando W, Langevin SM, Houseman EA, Nelson HH, Karagas MR, Wiencke JK, Kelsey KT (2012). Peripheral blood immune cell methylation profiles are associated with nonhematopoietic cancers. Cancer Epidemiol Biomark Prev.

[23] Wang Y, Li D, Li X, Teng C, Zhu L, Cui B, Zhao Y, Hu F (2014). Prognostic significance of hMLH1/hMSH2 gene mutations and hMLH1 promoter methylation in sporadic colorectal cancer. Med Oncol.

[24] Gupta S, Jaworska-Bieniek K, Narod SA, Lubinski J, Wojdacz TK, Jakubowska A (2014). Methylation of the BRCA1 promoter in peripheral blood DNA is associated with triple-negative and medullary breast cancer. Breast Cancer Res Treat.

[25] Chung W, Bondaruk J, Jelinek J, Lotan Y, Liang S, Czerniak B, Issa JP (2011). Detection of bladder cancer using novel DNA methylation biomarkers in urine sediments. Cancer Epidemiol Biomarkers Prev.

[26] Gao HL, Wang X, Sun HR, Zhou JD, Lin SQ, Xing YH, Zhu L, Zhou HB, Zhao YS, Chi Q. Methylation status of transcriptional modulatory genes associated with colorectal cancer in Northeast China. Gut Liver. 2018;12(2).. 10.5009/gnl17163 PMC5832342 29291617

[27] Wojdacz TK, Dobrovic A. Methylation-sensitive high resolution melting (MS-HRM): a new approach for sensitive and high-throughput assessment of methylation. Nucleic acids research. 2007;35(6):e41. 10.1093/nar/gkm013 PMC1874596 17289753

[28] Copeland KT, Checkoway H, McMichael AJ, Holbrook RH (1977). Bias due to misclassification in the estimation of relative risk. Am J Epidemiol.

