# Supplementary material for: Methylation of WT1, CA10 in peripheral blood leukocyte is associated with breast cancer risk: a case-control study
Source: BMC Cancer. 2020 Jul 31;20:713. doi: 10.1186/s12885-020-07183-8 (PMC7393705; doi:10.1186/s12885-020-07183-8)
Supplement: Supplementary file 1 — Additional file1 Table S1. Demographic variables and questionnaire-derived variables of participants before and after multiple imputation in this study. [file 12885_2020_7183_MOESM1_ESM.docx]

**Table S1** Demographic variables and questionnaire-derived variables of participants before and after multiple imputation in this study

| Variables |  | Before multiple imputation No. (%) | After multiple imputation No. (%) |
| --- | --- | --- | --- |
| Age | (mean ± SD) | 58.6(10.59) | 58.6(10.59) |
| BMI | ≤18.5 | 35(3.6) | 35(3.6) |
|  | 18.5- | 485(50.5) | 500(52.2) |
|  | ≥24.0 | 419(43.6) | 424(44.2) |
|  |  | 21(2.2) |  |
| Urban and Rural Status | Rural | 466(48.5) | 469(48.9) |
|  | Urban | 488(50.8) | 490(51.1) |
| Missing value |  | 6(0.6) |  |
| Education Level | Primary School or Below | 259(27.0) | 260(27.1) |
|  | Middle School | 310(32.3) | 311(32.3) |
|  | Senior School and Higher | 387(40.3) | 388(40.6) |
| Missing value |  | 4(0.4) |  |
| Ethnicity | Han | 910(94.8) | 915(95.3) |
|  | Other | 43(4.5) | 44(4.7) |
| Missing value |  | 7(0.7) |  |
| Mammography | No | 783(81.6) | 803(83.6) |
|  | Yes | 152(15.8) | 156(16.4) |
| Missing value |  | 25(2.6) |  |
| Breast Disease History | No | 730(76.0) | 735(76.6) |
|  | Yes | 222(23.1) | 224(23.4) |
| Missing value |  | 8(0.8) |  |
| Gynecologic Surgery | No | 592(61.7) | 593(61.8) |
|  | Yes | 362(37.7) | 366(38.2) |
| Missing value |  | 6(0.6) |  |
| Menstrual Cycle | Regular | 841(87.6) | 580(88.5) |
|  | Irregular | 106(11.0) | 109(11.5) |
| Missing value |  | 13(1.4) |  |
| Menopause | No | 423(44.1) | 427(44.5) |
|  | Yes | 526(54.8) | 532(55.5) |
| Missing value |  | 11(1.1) |  |
| Reproduction(times) | ≤1 | 503(52.4) | 522(54.4) |
|  | >1 | 423(44.1) | 437(45.6) |
| Missing value |  | 34(3.5) |  |
| Abortion(times) | ≤1 | 484(50.4) | 495(51.6) |
|  | >1 | 454(47.3) | 464(48.4) |
| Missing value |  | 22(2.3) |  |
| Breast Feeding | No | 63(6.6) | 68(7.2) |
|  | Yes | 856(89.2) | 891(92.8) |
| Missing value |  | 41(4.3) |  |

Continue

| Variables |  | Before multiple imputation No. (%) | After multiple imputation No. (%) |
| --- | --- | --- | --- |
| Oral Contraceptive | No | 843(87.8) | 850(88.5) |
|  | Yes | 109(11.4) | 109(11.5) |
| Missing value |  | 8(0.8) |  |
| Female Hormone Intake | No | 920(95.8) | 925(96.4) |
|  | Yes | 34(35) | 34(3.6) |
| Missing value |  | 6(0.6) |  |
| Fruit (g/week) | ≥500 | 450(46.9) | 461(48.7) |
|  | <500 | 488(50.8) | 498(51.3) |
| Missing value |  | 22(2.3) |  |
| Vegetable (g/week) | ≥500 | 424(44.2) | 428(44.6) |
|  | <500 | 526(54.8) | 531(55.4) |
| Missing value |  | 10(1.0) |  |
| Tomato (g/week) | ≥250 | 384(40.0) | 385(40.1) |
|  | <250 | 571(59.5) | 574(59.9) |
| Missing value |  | 5(0.5) |  |
| Broccoli (g/week) | ≥100 | 341(35.5) | 344(35.8) |
|  | <100 | 612(63.7) | 615(64.2) |
| Missing value |  | 7(0.7) |  |
| Beef and Lamb | Yes | 456(47.5) | 478(49.8) |
|  | No | 463(48.2) | 481(50.2) |
| Missing value |  | 41(4.3) |  |
| Bean Products(g/week) | ≥100 | 547(57.0) | 549(57.2) |
|  | <100 | 410(42.7) | 410(47.8) |
| Missing value |  | 3(0.3) |  |
| Pork (g/week) | <250 | 545(56.8) | 567(59.1) |
|  | ≥250 | 375(39.1) | 392(40.8) |
| Missing value |  | 40(4.2) |  |
| Chicken | No | 434(45.2) | 454(47.3) |
|  | Yes | 485(50.5) | 505(52.7) |
| Missing value |  | 41(4.3) |  |
| Seafood (g/month) | <250 | 499(52.0) | 503(52.5) |
|  | ≥250 | 454(47.3) | 456(47.5) |
| Missing value |  | 7(0.7) |  |
| Egg (eggs/week) | ≤3 | 405(42.2) | 407(42.5) |
|  | >3 | 550(57.3) | 552(57.5) |
| Missing value |  | 5(0.5) |  |
| Diary (times/week) | <2 | 479(49.9) | 505(52.6) |
|  | ≥2 | 425(44.3) | 454(47.3) |
| Missing value |  | 56(5.8) |  |
| Fungus (g/week) | ≥100 | 521(54.3) | 523(54.5) |
|  | <100 | 436(45.4) | 436(45.5) |
| Missing value |  | 3(0.3) |  |

Continue

| Variables |  | Before multiple imputation No. (%) | After multiple imputation No. (%) |
| --- | --- | --- | --- |
| Pickles (times/week) | <1 | 426(44.4) | 430(44.8) |
|  | ≥1 | 527(54.9) | 529(55.2) |
| Missing value |  | 7(0.7) |  |
| Tea | No | 815(84.9) | 520(85.4) |
|  | Yes | 138(14.4) | 139(14.5) |
| Missing value |  | 7(0.7) |  |
| Alcohol | No | 855(89.1) | 861(89.7) |
|  | Yes | 97(10.1) | 98(10.3) |
| Missing value |  | 8(0.8) |  |
| Cigarette | No | 833(86.8) | 838(87.3) |
|  | Yes | 119(12.4) | 121(12.7) |
| Missing value |  | 8(0.8) |  |
| Physical Activity | Yes | 578(60.2) | 582(60.6) |
|  | No | 374(39.0) | 377(39.4) |
| Missing value |  | 8(0.8) |  |
| Occupation Type | Blue Collar | 484(50.4) | 507(52.8) |
|  | White Collar | 432(45.0) | 452(47.2) |
| Missing value |  | 44(4.6) |  |
| Family History of  Breast Cancer | No | 926(96.5) | 928(96.7) |
|  | Yes | 30(3.1) | 31 (3.3) |
| Missing value |  | 4(0.4) |  |
| Family History of  Cancer | No | 746(77.7) | 748(77.9) |
|  | Yes | 210(21.9) | 211(22.1) |
| Missing value |  | 4(0.4) |  |
