# Supplementary material for: Methylation of WT1, CA10 in peripheral blood leukocyte is associated with breast cancer risk: a case-control study
Source: BMC Cancer. 2020 Jul 31;20:713. doi: 10.1186/s12885-020-07183-8 (PMC7393705; doi:10.1186/s12885-020-07183-8)
Supplement: Supplementary file 2 — Additional file2 Table S2. Primer sequences and reaction condition for methylation-sensitive high-resolution melting analysis. [file 12885_2020_7183_MOESM2_ESM.docx]

Table S2. Primer sequences and reaction condition for methylation-sensitive high-resolution melting analysis

| Gene name | Primer position | Primer sequence (5' to 3') | Cycling time(sec) | Annealing Temperature(°C) | Melting Temperature(°C) | °C/step | cycles |
| --- | --- | --- | --- | --- | --- | --- | --- |
| *WT1* | exon 1 | F-TATACGTGGAAGTCGGGTTTTGTA | 10 15 15 | 62-50 | 71-95 | 0.3 | 60 |
|  |  | R-CCAAAACCGAAACGACAACCCAAA |  |  |  |  |  |
| *CA10* | exon2 | F-GAGCGGGGAGATTTTTAAGGT | 10 30 20 | 64-61 | 65-90 | 0.3 | 55 |
|  |  | R-AAATTATTCCGACAAATCTCCCCT |  |  |  |  |  |
