# Supplementary material for: Methylation of WT1, CA10 in peripheral blood leukocyte is associated with breast cancer risk: a case-control study
Source: BMC Cancer. 2020 Jul 31;20:713. doi: 10.1186/s12885-020-07183-8 (PMC7393705; doi:10.1186/s12885-020-07183-8)
Supplement: Supplementary file 3 — Additional file3 Table S3. Reaction system for methylation-sensitive high-resolution melting analysis of WT1 and CA10. [file 12885_2020_7183_MOESM3_ESM.docx]

Table S3. Reaction system for methylation-sensitive high-resolution melting analysis of *WT1* and *CA10*

| Reagents | 1× | |
| --- | --- | --- |
|  | *WT1* | *CA10* |
| DNA(20ng/μL) | 0.8 | 0.8 |
| 2× LightCycler 480 High Resolution Melting Master Mix (μL) | 5 | 5 |
| Primer (10ng/μL) | 0.2 | 0.2 |
| Mg2+ (mM) | 1.6 | 1.5 |
| ddH2O (μL) | 2.4 | 2.5 |
| Total volume (μL) | 10 | 10 |
