# Supplementary material for: Methylation of WT1, CA10 in peripheral blood leukocyte is associated with breast cancer risk: a case-control study
Source: BMC Cancer. 2020 Jul 31;20:713. doi: 10.1186/s12885-020-07183-8 (PMC7393705; doi:10.1186/s12885-020-07183-8)
Supplement: Supplementary file 4 — Additional file4 Table S4. Result of methylation-sensitive high-resolution melting analysis for the same samples in different runs. [file 12885_2020_7183_MOESM4_ESM.docx]

Table S4 Result of methylation-sensitive high-resolution melting analysis for the same samples in different runs

| Gene | Sample type | No. | Result-1 | Result-2 |
| --- | --- | --- | --- | --- |
| *WT1* | Case | 476320 | Methylation | Methylation |
|  | Case | 476494 | Methylation | Methylation |
|  | Case | 488051 | Methylation | Methylation |
|  | Case | 449702 | Methylation | Methylation |
|  | Case | 460112 | Methylation | Methylation |
|  | Case | 503460 | Methylation | Methylation |
|  | Case | 448337 | Methylation | Methylation |
|  | Case | 473684 | Methylation | Methylation |
|  | Case | 472169 | Methylation | Methylation |
|  | Case | 520891 | Methylation | Methylation |
|  | Case | 521006 | Methylation | Methylation |
|  | Case | 517208 | Methylation | Methylation |
|  | Case | 519516 | Methylation | Methylation |
|  | Case | 492561 | Methylation | Methylation |
|  | Control | 31226 | Unmethylation | Unmethylation |
|  | Control | 31374 | Methylation | Methylation |
|  | Control | 31401 | Methylation | Methylation |
|  | Control | D12078 | Methylation | Methylation |
|  | Control | 41113 | Methylation | Methylation |
|  | Control | 41064 | Methylation | Methylation |
|  | Control | 41206 | Methylation | Methylation |
|  | Control | D12026 | Methylation | Methylation |
|  | Control | D12467 | Methylation | Methylation |
|  | Control | D12345 | Methylation | Methylation |
|  | Control | D11067 | Unmethylation | Unmethylation |
|  | Control | D12407 | Methylation | Methylation |
|  | Control | D12263 | Methylation | Methylation |
|  | Control | D12372 | Methylation | Methylation |
|  | Control | D12561 | Methylation | Methylation |
|  | Control | D13001 | Methylation | Methylation |
|  | Control | D13022 | Methylation | Methylation |
|  | Control | D13037 | Methylation | Methylation |
| *CA10* | Case | 476320 | Methylation | Methylation |
|  | Case | 476494 | Methylation | Methylation |
|  | Case | 488051 | Methylation | Methylation |
|  | Case | 449702 | Unmethylation | Unmethylation |
|  | Case | 460112 | Methylation | Methylation |
|  | Case | 503460 | Methylation | Methylation |
|  | Case | 448337 | Methylation | Methylation |
|  | Case | 473684 | Methylation | Methylation |
|  | Case | 472169 | Unmethylation | Unmethylation |
|  | Case | 520891 | Methylation | Methylation |
|  | Case | 521006 | Unmethylation | Unmethylation |
|  | Case | 517208 | Methylation | Methylation |
|  | Case | 519516 | Unmethylation | Unmethylation |
|  | Case | 518626 | Methylation | Methylation |
|  | Control | 31226 | Unmethylation | Unmethylation |
|  | Control | 31374 | Unmethylation | Unmethylation |
|  | Control | 31401 | Methylation | Methylation |
|  | Control | 41137 | Methylation | Methylation |
|  | Control | 41113 | Methylation | Methylation |
|  | Control | 41064 | Unmethylation | Unmethylation |
|  | Control | 41206 | Unmethylation | Unmethylation |
|  | Control | 31477 | Methylation | Methylation |
|  | Control | D12467 | Methylation | Methylation |
|  | Control | D12345 | Unmethylation | Methylation |
|  | Control | D11067 | Methylation | Methylation |
|  | Control | D12407 | Unmethylation | Unmethylation |
|  | Control | D12263 | Unmethylation | Unmethylation |
|  | Control | D12372 | Unmethylation | Unmethylation |
|  | Control | D12561 | Unmethylation | Unmethylation |
|  | Control | D13001 | Methylation | Methylation |
|  | Control | D13022 | Unmethylation | Unmethylation |
|  | Control | D13037 | Methylation | Methylation |
