# Supplementary material for: Methylation of WT1, CA10 in peripheral blood leukocyte is associated with breast cancer risk: a case-control study
Source: BMC Cancer. 2020 Jul 31;20:713. doi: 10.1186/s12885-020-07183-8 (PMC7393705; doi:10.1186/s12885-020-07183-8)
Supplement: Supplementary file 5 — Additional file5 Table S5. The methylation of WT1 and CA10 and clinicopathological characteristics in breast cancer patients. [file 12885_2020_7183_MOESM5_ESM.docx]

Table S5. The methylation of *WT1* and *CA10* and clinicopathological characteristics in breast cancer patients

| Clinicopathological  characteristics | | *WT1* | | | |  | *CA10* | | | |
| --- | --- | --- | --- | --- | --- | --- | --- | --- | --- | --- |
|  |  | No. of Unmethylation(%) | No. of Methylation(%) | Crude OR(95% CI) | *P* Value |  | No. of Unmethylation(%) | No. of Methylation(%) | Crude OR(95% CI) | *P* Value |
| TNM Stages | I | 4(16.7) | 101(28.1) | 1 |  |  | 32(27.8) | 73(27.1) | 1 |  |
|  | II | 16(66.7) | 224(62.2) | 0.52(0.16-1.71) | 0.28 |  | 70(60.9) | 170(63.2) | 1.09(0.66-1.80) | 0.74 |
|  | III & IV | 4(16.6) | 35(9.7) | 0.32(0.07-1.45) | 0.14 |  | 13(11.3) | 26(9.7) | 0.90(0.41-1.97) | 0.79 |
| Tumor Size | ≤2cm | 12(50.0) | 161(44.7) | 1 |  |  | 53(46.1) | 120(44.6) | 1 |  |
|  | >2cm | 12(50.0) | 199(55.3) | 1.13(0.47-2.69) | 0.79 |  | 62(53.9) | 149(55.4) | 1.08(0.69-1.67) | 0.74 |
| Lymph Nodes Involved | N0 | 10(41.7) | 184(51.1) | 1 |  |  | 63(54.8) | 131(48.7) | 1 |  |
|  | N1/N3 | 14(58.3) | 176(48.9) | 0.68(0.29-1.58) | 0.37 |  | 52(45.2) | 138(51.3) | 1.29(0.83-2.00) | 0.25 |
| Histological Type | Noninvasive | 2(8.3) | 45(12.5) | 1 |  |  | 18(15.7) | 29(10.8) | 1 |  |
|  | Invasive | 22(91.7) | 315(87.5) | 0.54(0.09-3.30) | 0.50 |  | 97(84.3) | 240(89.2) | 1.53(0.81-2.87) | 0.19 |
| ER Status | Negative | 8(33.3) | 96(26.7) | 1 |  |  | 39(33.9) | 65(24.2) | 1 |  |
|  | Positive | 16(66.7) | 264(73.3) | 1.28(0.51-3.23) | 0.60 |  | 76(66.1) | 204(75.8) | 1.57(0.99-2.57) | 0.06 |
| PR Status | Negative | 7(29.2) | 128(35.6) | 1 |  |  | 46(40.0) | 89(33.1) | 1 |  |
|  | Positive | 17(70.8) | 232(64.4) | 0.77(0.31-1.94) | 0.58 |  | 69(60.0) | 180(66.9) | 1.34(0.85-2.10) | 0.21 |
| HER2 Status | Negative | 5(20.8) | 124(34.4) | 1 |  |  | 39(33.9) | 90(33.5) | 1 |  |
|  | Positive | 19(79.2) | 236(65.3) | 0.45(0.15-1.33) | 0.15 |  | 76(66.1) | 179(66.5) | 1.04(0.65-1.65) | 0.87 |
| P53 | Negative | 16(66.7) | 271(75.3) | 1 |  |  | 83(72.2) | 204(75.8) | 1 |  |
|  | Positive | 8(33.3) | 89(24.7) | 0.71(0.28-1.78) | 0.46 |  | 32(27.8) | 65(24.2) | 0.81(0.49-1.33) | 0.40 |
